# Demographic and biologic influences on survival in whites and blacks: 40 years of follow-up in the Charleston heart study

**DOI:** 10.1186/1475-9276-5-8

**Published:** 2006-07-03

**Authors:** Paul J Nietert, Susan E Sutherland, Julian E Keil, David L Bachman

**Affiliations:** 1 Department of Biostatistics, Bioinformatics, and Epidemiology, Medical University of South Carolina, 135 Cannon St., Suite 303, P.O. Box 250835, Charleston, SC 29425, USA; 2 Mission Hospitals, Inc., Research Institute, 509 Biltmore Avenue, Asheville, NC 28801, USA; 3 Department of Neurosciences, Medical University of South Carolina, 96 Jonathan Lucas Street, P.O. Box 250606, Charleston, SC, USA

## Abstract

**Background:**

In the United States, life expectancy is significantly lower among blacks than whites. We examined whether socioeconomic status (SES) and cardiovascular disease (CVD) risk factors may help explain this disparity.

**Methods:**

Forty years (1961 through 2000) of all-cause mortality data were obtained on a population-based cohort of 2,283 subjects in the Charleston Heart Study (CHS). We examined the influence of SES and CVD risk factors on all-cause mortality.

**Results:**

Complete data were available on 98% of the original sample (647 white men, 728 white women, 423 black men, and 443 black women). After adjusting for SES and CVD risk factors, the hazard ratios (HRs) for white ethnicity were 1.14 (0.98 to 1.32) among men and 0.90 (0.75 to 1.08) among women, indicating that the mortality risk was 14% greater for white men and 10% lower for white women compared to their black counterparts. However the differences were not statistically significant.

**Conclusion:**

While there are marked contrasts in mortality among blacks and whites in the CHS, the differences can be largely explained by SES and CVD risk factors. Continued focus on improving and controlling cardiovascular disease risk factors may reduce ethnic disparities in survival.

## Background

Significant differences in life expectancy exist between whites and blacks in the United States. According to a 2004 report by the Nation Center for Health Statistics, the life expectancy for white females born in 2001 is 80.2 years, compared to 75.5 years in black females, 75.0 years in white males, and 68.6 years in black males.[1] Black Americans also exhibit higher rates of mortality from heart disease, cancer, cerebrovascular disease, and HIV/AIDS than any other ethnic group in the U.S.[2]

Much of the disparities have been attributed to cultural, societal, healthcare system, and environmental/geographic factors. [2-4]. Residential segregation, associated with each of these domains, has been reported as being the single most important contributor to adverse effects on socioeconomic status (SES) and the health of blacks in the U.S., as higher degrees of residential segregation have been associated with greater concentrations of poverty, lower quality of education, higher rates of crime (including homicide) and unemployment, and higher levels of environmental toxins.[5] Differential access to certain types of medical care may also explain a significant proportion of racial disparities in health, even after adjusting for SES.[6,7] By the restriction of socioeconomic opportunities and mobility, individual and institutional discrimination may also have adverse effects health.[5]

Individual characteristics are also purported to play a role in health disparities. Individual cardiovascular disease (CVD) risk factors and SES are particularly associated with survival.[3,8-11] Findings from the National Longitudinal Mortality Study showed that SES status accounted for 37%-67% of the black excess in mortality among women for accidents, ischemic heart disease, diabetes, and homicide, and 30%-50% of the black excess in mortality among men for accidents, lung cancer, stomach cancer, stroke, and homicide.[12] However, the associations between SES and health outcomes may be different for men and women. For example, although some research suggests that the association between SES and total mortality may be weaker among women than men,[13,14] an investigation of the First National Health and Nutrition Examination Survey found that the association between SES and risk of coronary heart disease may be stronger among women than among men.[15] These findings suggest the need for thorough, gender-specific analyses examining the effect of SES and race on survival.

Increased prevalence of CVD risk factors may be responsible for the excess mortality risk observed among blacks when compared to whites. For example, in the state economic areas surrounding the Atherosclerosis Risk in Communities (ARIC) study communities, blacks were shown to have higher blood pressure and glucose levels and a higher prevalence of obesity and diabetes.[3] The authors also reported finding a greater clustering of risk factors among blacks.

In the present study, we examine the extent to which SES and CVD risk factors account for ethnic disparities in mortality rates observed in men and women in the Charleston Heart Study (CHS), a population based prospective cohort study begun in 1960. An earlier published study of the CHS cohort comparing 28-year all-cause mortality rates between white and black men suggests that SES accounts for most of the observed ethnic disparity in mortality among men,[10] as did a 30-year follow-up analysis of both men and women in the CHS cohort.[16] The present investigation extends the follow-up of this cohort for an additional 10-12 years and also includes data from women. The CHS provides an excellent opportunity to examine the influence of SES in a population-based cohort that contains a substantial proportion of blacks of varied SES and key baseline CVD risk factors, over a 40 year period.

## Materials and methods

The CHS originally included a population-based sample of 2,181 adults over age 35, representative of residents of Charleston County, SC. In 1963, a sample of 102 peer-nominated, black men of high SES was added to the cohort. The peer nomination process involved investigator interviews with black men who were prominent in the community (business owners, professionals, etc.) who, in turn, identified other high SES black men in Charleston County. Because blacks and whites comprised over 99% of the ethnic composition of the Charleston County population during this time period, no other ethnic groups were included. Details of the baseline examination and sampling plan were published earlier.[17] The sampling plan originally involved an expected sample of 2,500 subjects drawn from the 1950 US Census, approximately 3.825% of the county's population at that time. Units of 12 households were randomly selected, and all adults 35 years and older were approached for participation, with an 84% response rate.[17]

Data gathered at baseline (1960 for population-based sample, 1963 for high SES black men) included age, gender, ethnicity (white/black), years of education, occupation, self-reported history of diabetes, current and past smoking status, and measurement of height, weight, total serum cholesterol, and 2 blood pressure readings. For the purposes of this analysis, occupational categories were dichotomized into "blue collar" and "non-blue collar" based upon the original (1960) classification categories. Occupation was re-categorized as "blue collar" from the original designations of: farm laborer/foreman; laborer except farm/household help; craftsman, foreman or kindred worker; or operative worker. Jobs that were not considered blue collar included professionals; military service personnel; farm managers; proprietors, managers and officials (non-farm); clerical, sales and kindred workers; and service/protective service workers. If a person was retired, his or her usual occupation prior to retirement was used. Since many women (50.8%) did not report a usual occupation, their occupational status was dichotomized as employed or unemployed. The two systolic blood pressure measurements were averaged, as were the two diastolic blood pressure measurements. Subjects were categorized as having elevated blood pressure at baseline if their systolic average was at least 140 mmHg or if their diastolic average was at least 90 mmHg. Body mass index (BMI) was derived from the recorded height and weight for each individual.

From the baseline examination through 1995, participants were examined 6 times, with vital status updated routinely. A recent update in vital status was conducted using the National Center for Health Statistics National Death Index and the Social Security Death index[18] to obtain 40 years of mortality data. The vital status is known on 98% of the cohort for the period 1960-2000. Using linear and logistic regression models to adjust for age in 1960, baseline characteristics were compared between white men and both the general population of black men and the high SES black men, and between white and black women. This study was approved by the Institutional Review Board of the Medical University of South Carolina.

Kaplan-Meier curves were created to illustrate the observed survival rates for each group, and log-rank tests were used to assess differences in unadjusted survival times across the gender/ethnicity strata. A series of Cox proportional hazards regression models were then used to determine whether there was significant variation in survival rates by ethnicity. Proportional hazards models were used because they allow for the estimation of the relative risk of mortality while adjusting for other variables. In all survival models, time to death (or time to last contact for survivors) served as the dependent variable. If no death was recorded for a given individual subject, the date of last contact served as the censoring date. In order to determine whether ethnicity was significantly and independently associated with time to death, initial analyses included an assessment of the interaction between ethnicity and gender. The ethnicity by gender interaction was found to be significant (p < 0.01); thus subsequent models were stratified by gender.

To quantify the unadjusted ethnic disparity in observed survival, the first gender-specific models only included ethnicity and baseline age as dependent variables. To demonstrate the effect of SES on survival, a second series of models included education and occupation in addition to the dependent variables included in the first series of models. Lastly, the full models (series 3) included all baseline variables and any significant 2-way interactions involving ethnicity. A stepwise procedure was used to allow interactions to enter the model if the significance level associated with their inclusion was less than 0.15, and interactions were allowed to remain in the model only if their final level of significance was less than 0.05. However, for both men and women, none of the interactions between ethnicity and any of the baseline covariates were significant, and thus none were included in the final models. The proportional hazards assumption was verified for each of the full models presented. Because income was not available at baseline, we performed a similar survival analysis on 933 (41%) of the 2,283 original participants that included the 1987/1989 income measure. These analyses allowed us to determine if the inclusion of income would have significantly altered our findings. To examine whether survival patterns in Charleston County may be deemed generalizable to other areas of the country, an additional analysis was performed to compare the observed deaths in the CHS cohort to the expected number of deaths had the CHS cohort died at rates (obtained from NCHS[19]) observed throughout the entire United States during this time frame.

## Results

Complete data were available on 2,241 (98%) of the original 2,283 participants, and the median follow-up time was 27 years. Table 1 summarizes the baseline comparisons in the cohort. Blacks averaged significantly less formal education than whites, although the highest levels of education were observed among the high SES black men. White men were significantly less likely than the general population of black men to work in blue collar settings, although almost all high SES black men did not work in blue collar jobs. White women were significantly less likely than black women to be employed. Among men, smoking was more prevalent among the general population of blacks than whites, although high SES black men were the least likely to smoke. Smoking rates were similar among white and black women. Among men, cholesterol levels were highest among the high SES black men, followed by white men, and the general population of black men. Cholesterol was higher among white women than among black women. The highest rates of self-reported diabetes occurred in the high SES black men and in black women. Higher blood pressures were observed among both groups of black men compared to white men, and among black women compared to white women. The high SES black men had significantly higher BMIs than white men, and black women had significantly higher BMIs than white women.

**Table 1:** Baseline characteristics of study sample

	Population Sample Group				
	White Men	Black Men^†^	High SES Black Men	White Women	Black Women
Variable	N = 647	N = 321	N = 102	N = 728	N = 443
Age in 1960 (years: mean [s.d.])	49.9 (10.9)	49.9 (11.9)	42.3 (10.8)*	50.1 (11.4)	50.4 (12.4)
Education (years: mean [s.d.])^††^	10.0 (3.7)	4.9 (3.4)*	14.7 (3.2)*	10.0 (3.1)	5.6 (3.6)*
Occupational Status:^††^					
Blue Collar	52.1%	93.4%*	1.0%*	-	-
Employed outside the home	-	-	-	38.7%	66.3%*
Smoking history^††^					
Current smoker (%)	69.4%	76.3%*	49.0%*	39.7%	37.9%
Former smoker (%)	17.6%	7.2%*	18.6%	8.2%	7.5%
Cholesterol (mg/Dl: mean [s.d.])^††^	237 (45)	221 (45)*	249 (59)*	242 (52)	234 (48)*
Hx Diabetes^††^	3%	2%	6%*	2%	6%*
SBP (mmHg: mean [s.d.])^††^	139.2 (22.8)	151.2 (29.4)*	139.1 (20.2)*	137.8 (25.5)	159.9 (34.2)*
DBP (mmHg: mean [s.d.])^††^	83.8 (10.0)	90.3 (14.3)*	85.3 (12.4)*	82.0 (10.2)	91.9 (13.7)*
Elevated blood pressure^††^	42.7%	62.5%*	48.0%*	39.3%	69.4%*
BMI (mean [s.d.])^††^	25.1 (3.7)	25.1 (4.4)	26.7 (4.5)*	24.4 (4.6)	27.3 (6.3)*

SES: socioeconomic status; SBP: systolic blood pressure; DBP: diastolic blood pressure; BMI: body mass index

* p < 0.05 compared to the white population of the same gender

^† ^The group labeled as 'Black Men' refers to the general population of black men recruited in 1960 and does not include the group of high SES black men recruited in 1963.

^†† ^All comparisons adjusted for age in 1960, using an analysis of covariance model or a logistic regression model, as appropriate.

**Figure 1:**
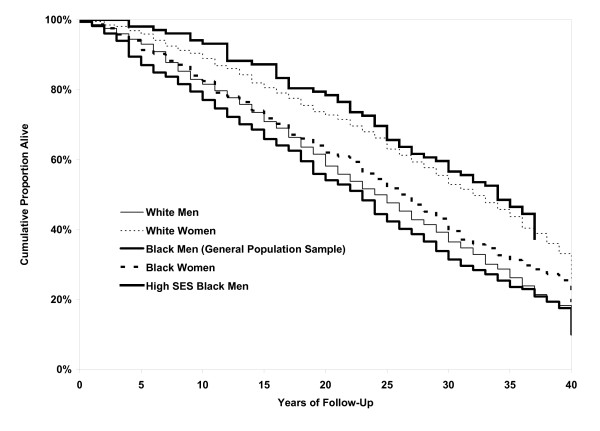
Kaplan-Meier survival curves for the 5 population groups of the Charleston Heart Study.

Figure 1 shows the Kaplan-Meier survival curves for the 5 population groups of interest. The log-rank tests indicated highly significant unadjusted differences in survival across these 5 groups (chi-square = 79.4 [4df], p < 0.0001). Differences in survival between white and black men (general population sample only) were not significantly different (p = 0.28); however the differences between black and white women were highly significant (p < 0.001). Median years of follow-up were 24 years for white men, 23 years for black men, 34 years for high SES black men (note, however, that they were younger at baseline than the other groups of men), 32 years for white women, and 26 years for black women. The median observed ages at death were: 77 years for white men, 74 years for black men, 78 years for high SES black men, 83 years for white women, and 78 years for black women.

A summary of the Cox proportional hazards models for men is shown in Table 2. Hazard ratios greater than 1 indicate an increased mortality rate (i.e. an increased risk of dying at any given point in time conditioned upon survival to the beginning of that time point) and thus an decreased probability of survival during any specified time interval, while hazard ratios less than 1 indicate a decreased mortality rate, or an increased probability of survival during any specified time interval. The first model indicates that the age-adjusted ethnicity effect on survival was not significant. As was the case in this model and all subsequent models, the effect of baseline age was highly significant (p < 0.001), with older subjects exhibiting an increased hazard compared to their younger counterparts. When education and occupation were introduced into the model (i.e. model 2), we observed that more highly educated subjects exhibited increased probability of survival during the study time frame. Additionally, the ethnicity effect among men appeared stronger than in model 1, with black men demonstrating increased survival rates when compared to white men. Results from the full model (model 3) suggest that survival rates were significantly worse among men who at baseline were older (p < 0.0001), were current smokers (p < 0.0001), had diabetes (p < 0.001), or who had elevated blood pressure (p < 0.0001) when adjusted for all the other factors in the model. Among men, there was a trend (p = 0.060) for higher levels of education to be associated with improved survival, and no association was noted between all-cause mortality and ethnicity or a number of other baseline characteristics (i.e. blue collar occupation, being a former smoker, body mass index, or total serum cholesterol).

**Table 2:** Hazard ratios (HR) and 95% confidence intervals (CI) obtained from Cox proportional hazards models predicting time to death among men

	Model 1		Model 2		Model 3	
Baseline characteristic	HR	95% CI	HR	95% CI	HR	95% CI
White ethnicity	1.00	0.88, 1.16	1.10	0.95, 1.27	1.14	0.98, 1.32
Baseline age (years)	1.08	1.07, 1.09	1.08	1.07, 1.08	1.07	1.07, 1.08
Blue collar occupation	-		1.13	0.94, 1.34	1.08	0.90, 1.29
Education (years)	-		0.98	0.96, 1.00	0.98	0.96, 1.00
Current smoker	-		-		1.67	1.37, 2.03
Former smoker	-		-		1.23	0.96, 1.58
BMI (kg/m^2^)	-		-		0.99	0.97, 1.01
Total serum cholesterol*	-		-		1.00	0.98, 1.01
History of diabetes	-		-		1.84	1.29, 2.64
Elevated blood pressure**	-		-		1.41	1.22, 1.62

* The hazard ratio associated with total serum cholesterol is for a 10 mg/Dl increase.

** See text for elevated blood pressure definition.

Table 3 lists the results from the series of Cox proportional hazards models for women. The results of the first model indicate that white women appear to have significantly (p < 0.001) increased survival rates when compared to black women. Even when education and occupation were introduced into the model (i.e. model 2), the adjusted survival rates were significantly (p < 0.05) higher among white women, although the ethnicity effect was attenuated. The full model (model 3), however, indicates that among women in the CHS, survival rates were comparable (i.e. not statistically different) between whites and blacks (HR: 0.90, 95% CI: 0.75, 1.08) when other factors were considered. Among women, decreased survival rates were significantly associated with most of the baseline covariates: older age (p < 0.0001), being a current (p < 0.0001) or former (p < 0.05) smoker, higher BMI (p < 0.01), higher total serum cholesterol (p < 0.01), history of diabetes (p < 0.001), and elevated blood pressure (p < 0.0001). There was a trend for decreased survival rates to be associated with lower levels of education (p = 0.054), and no significant association was observed between mortality and employment status.

**Table 3:** Hazard ratios (HR) and 95% confidence intervals (CI) obtained from Cox proportional hazards models predicting time to death among women

Baseline characteristic	Model 1		Model 2		Model 3	
	HR	95% CI	HR	95% CI	HR	95% CI
White ethnicity	0.70	0.61, 0.80	0.81	0.68, 0.96	0.90	0.75, 1.08
Baseline age (years)	1.09	1.08, 1.10	1.09	1.08, 1.10	1.09	1.08, 1.09
Employed	-		0.98	0.85, 1.13	0.98	0.86, 1.13
Education (years)	-		0.97	0.95, 0.99	0.98	0.96, 1.00
Current smoker	-		-		1.40	1.21, 1.61
Former smoker	-		-		1.35	1.05, 1.74
BMI (kg/m^2^)	-		-		1.02	1.01, 1.03
Total serum cholesterol*	-		-		1.02	1.01, 1.04
History of diabetes	-		-		1.81	1.31, 2.51
Elevated blood pressure**	-		-		1.42	1.21, 1.66

* The hazard ratio associated with total serum cholesterol is for a 10 mg/Dl increase.

** See text for elevated blood pressure definition.

In the analyses of the 933 participants who provided income data in the 1987/89 interview, the hazard ratio (HR) comparing mortality between white to black men did not change substantially (model with no income: HR: 1.14, 95% CI: 0.98, 1.32; model with income: HR: 1.11, 95% CI: 0.83, 1.47), nor did it change substantially among women (model with no income: HR: 0.90, 95% CI: 0.75, 1.08; model with income: HR: 0.95, 95% CI: 0.67, 1.34). Given that the black members of the CHS cohort were generally of lower SES and had worse CVD risk factor profiles, adjustment for other key unmeasured baseline variables using data from later interviews/exams would probably only serve to further reduce the observed ethnic disparities in survival.

The standardized mortality ratios (SMR) of observed to expected numbers of deaths were all not significantly different from 1 for whites and the general population of blacks (white men: SMR: 0.97, 95% CI: 0.89, 1.05; white women: SMR: 0.92, 95% CI: 0.84, 1.00; black men: SMR: 0.97, 95% CI: 0.85, 1.08]; black women: SMR: 1.11, 95% CI: 0.99, 1.23). The high SES black men died at a much lower rate than would have been expected for black men in the United States (SMR: 0.56, 95% CI: 0.42, 0.70).

## Discussion

There are ethnic differences in the observed survival rates in the general population sample of the CHS cohort (i.e. excluding the high SES black men), primarily among women. In this sample, the median age at death was 3 years younger among black men when compared white men and 5 years younger among black women when compared white women. Among all men, after the inclusion of SES characteristics and traditional CVD risk factors, the effect of ethnicity on survival times diminished substantially. In fact, among men it was shown that whites had lower, albeit not significantly, adjusted survival rates than blacks. Among women, the unadjusted model indicated marked ethnic disparities in survival times, an effect that was attenuated with the addition of SES characteristics into the analyses (i.e. model 2). When certain baseline comorbid conditions (i.e. diabetes, elevated blood pressure) were included (i.e. model 3), the effect of ethnicity on survival times was further reduced, suggesting that ethnic disparities in CVD and associated comorbidity explains ethnic disparities in survival times among women.

These findings are consistent with earlier reports from the Charleston Heart Study[10,16] and from the Evans County (Georgia) Heart Study[11] cohort of ethnic comparisons of survival rates adjusted for SES. A 30-year follow-up study of the CHS demonstrated[16] after adjusting for baseline characteristics (including SES), that the black:white mortality ratio was 1.0 for men and 1.1 for women. The Evans County study showed that mortality risk ratios (RR) were only slightly higher among black men when compared to high SES white men in 2 age groups (40-64: RR = 1.19; = 65: RR = 1.00), and slightly lower when compared to low SES white men (40-64: RR = 0.92; = 65: RR = 0.90). In this Evans County study, they found that black women had significantly greater mortality risk ratios when compared to white women (RR = 1.39); however they did not adjust for baseline CVD risk factors.[11] A combined analysis of the CHS and Evans County cohorts also demonstrated that after adjustment for CVD risk factors, black men experienced mortality rates similar to that among white men.[20]

A study of ethnic disparities in 3-year fatality rates following hospitalization for myocardial infarction (MI) in the Atherosclerosis Risk in Communities study also demonstrated that SES has a significant attenuating effect on the influence of ethnicity.[21] In that study, a much larger proportion of black MI patients (21%) died within 3 years compared to white MI patients (14%). The age and sex adjusted relative hazard (RH) was significant (RH: 1.80, 95% CI: 1.24, 2.61); however, after additionally adjusting income or vascular risk factors, the RH was no longer significant (age, sex, and income adjusted RH: 1.31, 95% CI: 0.83, 2.09; age, sex, and vascular risk factor adjusted RH: 1.29, 95% CI: 0.83, 2.00). When the model accounted for SES, vascular risk factors, and in-hospital MI treatment procedures, there was no disparity (RH: 1.0, 95% CI: 0.56, 1.77). The authors conclude that better treatment following an MI could significantly reduce the differential fatality rates.

Although this current study showed that the effect of ethnicity on survival rates is substantially diminished when socioeconomic status is taken into consideration, it should be recognized that this finding does not necessarily indicate that there are no disparities in certain cause-specific mortality rates. For example, black men with prostate cancer have been shown in multivariate analyses to survive 1.7 years less, on average, than white men,[22] and black women with breast cancer experience higher mortality rates than white women.[23] Ethnic disparities have also been reported to exist for a variety of other causes of death, including coronary heart disease[24,25] and stroke.[26] Which causes of death are most influenced by socioeconomic status remain to be examined.

Socioeconomic status impacts survival rates for several reasons. People with low incomes are less likely to have health insurance and less likely to have access to primary and specialty healthcare, factors that are known to be closely associated with health outcomes. For example, children who live in poverty are more likely to receive lower-quality healthcare and to die in infancy.[27] In another study, poverty was correlated with higher rates of preventable hospitalization in an examination of California hospital discharge data, and uninsured persons experienced greater difficulty than privately insured patients in accessing inpatient care.[28] Education and income levels have also been shown to be associated with health behaviors (e.g. smoking, alcohol drinking, sedentary lifestyle, relative body weight); however studies suggest that socioeconomic differences in mortality rates would still persist to a significant extent even with improved health behaviors among people of lower socioeconomic status.[29,30]

Our study does have several limitations. There are several key SES indicators and CVD risk factors which were not measured on all participants at baseline in the CHS cohort, including income, access to healthcare services, serum/urine glucose levels, more detailed lipid measurements (e.g. high density lipoprotein, low density lipoprotein, triglycerides) or genetic markers (e.g. apolipoprotein E). Including the peer-nominated high SES cohort of black men may have introduced some selection bias into this study, given that these men may have been significantly healthier at baseline; thus it is possible that such a bias may have masked to some extent a true racial disparity among men. Other limitations include reliance upon self-reporting of diabetes versus confirmed diagnosis and not accounting for changes in risk factors over time. For example, although baseline total serum cholesterol was not significantly associated with survival rates among men, this does not necessarily mean that men's total cholesterol levels do not influence their risk of death. Cholesterol values could have increased or decreased over time among these men, thus affecting their risk of mortality.

A large proportion of the observed ethnic disparities in life expectancy are not able to be addressed in these analyses, namely the differences in mortality occurring before age 35. Recent life tables published by the National Center for Health Statistics (NCHS) suggest that 96.7% of white males will survive to age 35, compared to only 93.7% of black males. Similarly, 98.3% of white females will survive to age 35, compared to only 96.7% of black females.[1] By applying the white mortality rates to blacks under age 35, we determined that these disparities may account for as much as 1.6 years (25%) of the 6.4 year differential among life expectancy in white and black men and as much as 1.0 years (21%) of the 4.7 year differential among life expectancy in white and black women. While our study has shown that much of the observed ethnic disparity in life expectancy may be attributable to SES and certain CVD risk factors, there are clearly many other factors that contribute to the overall life expectancy disparities that influence people before age 35.

The findings of our study have several implications. While there are ethnic disparities in survival rates in the CHS cohort, particularly among women, much may be attributed to several preventable and/or modifiable risk factors including education, smoking, total cholesterol, blood pressure, body mass index, and diabetes. Since much of the observed ethnic disparities in survival can be explained by these factors, and since better control of cardiovascular disease risk factor leads to improved health outcomes, [31-33] continued focus on improving and controlling these cardiovascular disease risk factors may ultimately reduce ethnic disparities in mortality.

## Abbreviations

ARIC: Atherosclerosis Risk in Communities

CHS: Charleston Heart Study

CVD: cardiovascular disease

HR: hazard ratio

NCHS: National Center for Health Statistics

RH: relative hazard

RR: relative risk

SC: South Carolina

SES: socioeconomic status

## Competing interests

The author(s) declare that they have no competing interests.

## Authors' contributions

PJN participated in the design of the study, conducted the analyses, and drafted the manuscript. SES participated in the design of the study, helped provide advice on conducting the analyses, and helped draft the manuscript. JEK participated in the design of the study, helped provide advice on conducting the analyses, and helped draft the manuscript. DLB participated in the design of the study, helped provide advice on conducting the analyses, and helped draft the manuscript.

All authors read and approved the final manuscript.
